# Carvacrol Selective Pressure Allows the Occurrence of Genetic Resistant Variants of *Listeria monocytogenes* EGD-e

**DOI:** 10.3390/foods11203282

**Published:** 2022-10-20

**Authors:** Daniel Berdejo, Elisa Gayán, Elisa Pagán, Natalia Merino, Raúl Campillo, Rafael Pagán, Diego García-Gonzalo

**Affiliations:** Departamento de Producción Animal y Ciencia de los Alimentos, Facultad de Veterinaria, Instituto Agroalimentario de Aragón-IA2 (Universidad de Zaragoza-CITA), 50013 Zaragoza, Spain

**Keywords:** carvacrol, evolution assays, resistant variants, *Listeria monocytogenes*, heat treatments, antibiotic resistance, whole-genome sequencing

## Abstract

Essential oils and their constituents, such as carvacrol, are potential food preservatives because of their great antimicrobial properties. However, the long-term effects of these compounds are unknown and raise the question of whether resistance to these antimicrobials could emerge. This work aims to evaluate the occurrence of genetic resistant variants (RVs) in *Listeria monocytogenes* EGD-e by exposure to carvacrol. Two protocols were performed for the RVs selection: (a) by continuous exposure to sublethal doses, where LmSCar was isolated, and (b) by reiterative exposure to short lethal treatments of carvacrol, where LmLCar was isolated. Both RVs showed an increase in carvacrol resistance. Moreover, LmLCar revealed an increased cross-resistance to heat treatments at acid conditions and to ampicillin. Whole-genome sequencing identified two single nucleotide variations in LmSCar and three non-silent mutations in LmLCar. Among them, those located in the genes encoding the transcriptional regulators RsbT (in LmSCar) and ManR (in LmLCar) could contribute to their increased carvacrol resistance. These results provide information regarding the mode of action of this antimicrobial and support the importance of knowing how RVs appear. Further studies are required to determine the emergence of RVs in food matrices and their impact on food safety.

## 1. Introduction

Increasing restrictions on the use of chemically synthesized food preservatives, as well as consumer rejection of those additives, have led to the search for new food preservatives of natural origin [1]. In this regard, plant essential oils (EOs) and their individual constituents (ICs) have been proposed as alternatives to chemical preservatives used in the food industry [2] due to their great antimicrobial and antioxidant properties, among others [3,4].

However, the use of EOs and ICs for food preservation currently shows some drawbacks that compromise their industrial application. The necessary doses of EOs and ICs for food preservation are not very high, but even so, these small concentrations can cause food alterations in odor and taste that might be rejected by consumers [5]. Reducing the necessary doses for food preservation to concentrations that are not unpleasant or detectable by the consumer requires, among things, the knowledge of the mechanisms of action of these natural antimicrobials [6,7].

On the other hand, in recent decades, the emergence of bacterial antimicrobial resistance (AMR) and its spread in the environment has become one of the greatest hazards to global health [8,9]. One of the solutions to this problem focuses on the search for new antimicrobial compounds as an alternative to antibiotics currently used in human and veterinary medicine [10,11], such as EOS and ICs [12]. However, the long-term effects of EOs and ICs are unknown and raise the question of whether resistance to these natural antimicrobials could also appear. According to de Souza [13], the emergence of genetic resistant variants (RVs) by exposure to EOs and ICs would be unlikely. This fact would be due to the antioxidant capacity of these compounds at low doses [14], which can, in some cases, even reduce the mutagenic rate in Gram-negative [15] and Gram-positive bacteria [16]. Nevertheless, recent studies have shown that prolonged exposure to these natural antimicrobials can lead to the emergence of RVs in bacteria such as *Escherichia coli* [15], *Salmonella* Typhimurium [17], *Staphylococcus aureus* [16,18], and more recently, *Listeria monocytogenes* [19], that are insensitive not only against these compounds (direct-resistance) but also against antibiotics (cross-resistance) used in human and veterinary medicine [20].

The emergence of these RVs may pose a risk to food safety as they can survive lethal preservation treatments or grow under unfavorable conditions. In addition, the fact that RVs also exhibit development of cross-resistance to antibiotics may compromise the clinical treatment of RV-infections [20,21]. For this reason, it is necessary to determine under which conditions RVs to natural antimicrobials can appear in the food chain. Furthermore, the genetic study of these RVs to natural antimicrobials would allow the identification of the mutations responsible for resistance and, consequently, help target cellular structures or functions involved in the bacterial response to EOs and ICs. Consequently, this study might provide information on the mechanisms of action of these compounds. These results would make it possible to optimize the conditions of use of EOs and CIs, reducing the doses added to foods and facilitating sensory acceptance by the consumer.

Therefore, this study aims (a) to isolate RVs in *L. monocytogenes* by two evolution assays in the presence of carvacrol: one by continuous exposure to sublethal doses and another by exposure to short lethal treatments, (b) to characterize the direct resistance of the isolated RVs to carvacrol, (c) to assess the cross-resistance to heat treatments and antibiotics in the clinical use of the isolated RVs, and (d) to identify the genetic modifications that may be responsible for their increased resistance.

## 2. Materials and Methods

### 2.1. Microorganisms and Growth Conditions

*L. monocytogenes* EGD-e was kindly provided by Prof. Chakraborty (Institute for Medical Microbiology, Giessen, Germany). This bacterium has been extensively studied, including its whole-genome sequence [22,23,24], due to its high relevance in food outbreaks. Throughout this investigation, the strain was kept in cryovials at −80 °C with glycerol (20% *v*/*v*), from which plates of tryptone soya agar (Oxoid, Basingstoke, England) with 0.6% yeast extract (Oxoid; TSAYE) were prepared on a weekly basis. To prepare the working bacterial cultures, test tubes containing 5 mL of tryptone soya broth (Oxoid) with 0.6% yeast extract (TSBYE) were inoculated with one colony and then incubated aerobically overnight on an orbital shaker (130 rpm; Heidolph Vibramax 100, Schwabach, Germany) at 37 °C (Incubig, Selecta, Barcelona, Spain). Subsequently, flasks containing 10 mL of fresh TSBYE were inoculated with the resulting subculture to achieve an initial concentration of 10^6^ colony-forming units per mL (CFU/mL), which were incubated for 24 h at 37 °C and 130 rpm until the stationary growth phase was reached (2 × 10^9^ CFU/mL approximately). The same protocol was followed to obtain the bacterial cultures of the isolated strains that resulted from the evolution assays with carvacrol (≥98%; Sigma-Aldrich, Steinheim, Germany).

### 2.2. Minimum Inhibitory Concentration (MIC) and Minimum Bactericidal Concentration (MBC)

The minimum inhibitory concentration (MIC) was determined according to CLSI [25] with some modifications due to the hydrophobicity of the EO. First, strains were inoculated in test tubes with 5 mL of cation-adjusted Mueller–Hinton broth (Sigma-Aldrich; MHB) at an initial concentration of 5 × 10^5^ CFU/mL in the presence of different concentrations of carvacrol: from 50 up to 500 µL/L with 25 μL/L intervals (based on previous results not shown), which were incubated at 37 °C for 24 h and 130 rpm. Afterwards, MIC was determined as the lowest concentration of the antimicrobial compound that was capable of inhibiting bacterial growth. To objectively determine bacterial growth, the optical density was read at 595 nm (OD_595_) using a microplate reader (Genios, Tecan, Männedorf, Switzerland). In total, 10% of the OD_595_ measure of the positive control was established as the lower limit to consider that a strain could grow [26]. Vigorous shaking by vortex (Genius 3, Ika, Königswinter, Germany) was used to prepare carvacrol dispersions in MHB, thus avoiding the use of solvents that could be detrimental to antibacterial activity. Positive control tubes with 5 mL MHB inoculated at 5 × 10^5^ CFU/mL without ICs, and negative control tubes with 5 mL of MHB with 500 µL/L of carvacrol, were also prepared in every experiment.

In parallel, minimum bactericidal concentration (MBC) was evaluated. From the test tubes employed for the determination of MIC, a 100-µL aliquot of each tube was spread onto cation-adjusted Mueller–Hinton agar plates (Sigma-Aldrich; MHA), which were incubated at 37 °C for 48 h. Colonies were counted, and the lowest concentration of carvacrol that killed ≥99.9% of the initial bacterial population (5 × 10^5^ CFU/mL) was defined as the MBC endpoint [27]. The same positive and negative controls of the MIC test were employed in this experiment.

MIC and MBC were firstly determined in the wild-type strain to establish the necessary doses of carvacrol to be added in the evolution assays and then to compare the resistance of the evolved strains with that of the wild-type strain.

### 2.3. Carvacrol Evolution Assays

The wild-type *L. monocytogenes* strain (LmWT) was exposed to two different evolution assays to obtain RVs: (a) exposure to prolonged sublethal treatments and (b) cyclic exposure to short lethal treatments, according to Berdejo et al. [28].

(a) The first protocol was based on the isolation of strains by prolonged exposure to a subinhibitory concentration of carvacrol during bacterial growth. LmWT was grown on TSAYE plates for 48 h at 37 °C. A single colony was inoculated in 5 mL of TSBYE and incubated under agitation for 12 h at 37 °C. This preculture was diluted 1:1000 into 50 mL of TSBYE and incubated for 3.5 h to obtain an exponential phase culture. From this culture, 5 mL of TSBYE were inoculated at an initial bacterial concentration of 10^6^ CFU/mL in the presence of 75 µL/L of carvacrol (1/2 × MIC). This bacterial suspension was incubated (24 h/37 °C/130 rpm), and once the stationary phase was reached, the same step was repeated: the culture was diluted (up to 10^6^ CFU/mL) in 5 mL of TSBYE with 75 µL/L of carvacrol and incubated (24 h/37 °C/130 rpm). This procedure was repeated 20 times. After the 20th step, an aliquot was diluted in phosphate-buffered saline (Sigma-Aldrich; PBS) and spread on TSAYE plates (without carvacrol). After plate incubation for 48 h at 37 °C, five colonies were randomly picked to evaluate their MIC against carvacrol. Since the MIC values were similar for the five strains, one evolved clone was selected, hereinafter referred to as LmSCar, to carry out its phenotypic and genotypic characterization. This methodology was adapted from Kohanski, DePristo and Collins [26], and Andersson and Hughes [29].

(b) The second protocol was based on the isolation of strains by recovering surviving cells after short-term lethal treatments with carvacrol. For this purpose, a stationary phase culture of LmWT was diluted 1:100 in 50 mL of TSBYE with 300 µL/L of carvacrol (2 × MIC) and incubated for 4.5 h at 37 °C. Subsequently, treated cells were centrifuged for 20 min at 15,000 RCF (centrifuge 1736R, Gyrozen, Gimpo, South Korea), washed twice with TSBYE, resuspended in 1 mL of TSBYE, and incubated 19.5 h at 37 °C under agitation until the stationary phase was reached. This procedure was repeated 30 times. This assay had to be extended for 10 more days than protocol (a) in order to evolve the strain on the basis of its resistance. After the 30th step, an aliquot was diluted in PBS, spread on TSAYE plates (without carvacrol), and incubated for 48 h at 37 °C. After the incubation period, five colonies were randomly picked to evaluate their MIC against carvacrol. Since the MIC values were similar for the five strains, one evolved clone was selected, hereinafter referred to as LmLCar, to carry out phenotypic and genotypic characterization. This methodology was adapted from Levin-Reisman et al. [30].

### 2.4. Survival Curves to Lethal Concentrations of Carvacrol and Heat Treatments

The resistance of LmWT and evolved strains, LmSCar and LmLCar, against carvacrol was evaluated with lethal treatments. The treatment medium was citrate–phosphate buffer or “McIlvaine buffer” at pH 4.0 or pH 7.0 [31], prepared from citric acid monohydrate (Panreac) and disodium hydrogen phosphate (Panreac). Those pH values were chosen as representative of acidic and neutral foods. The treatment was carried out in 10 mL of McIlvaine buffer at 25 °C, to which carvacrol was added at a concentration of 200 µL/L, for treatment at pH 4.0, and 300 µL/L, for treatment at pH 7.0, and then vigorously agitated to obtain a homogeneous dispersion of the IC. This concentration was selected based on preliminary experiments where carvacrol treatment reached 5 log_10_ cycles of inactivation of LmWT. Once carvacrol was added, the stationary phase culture was centrifuged for 5 min at 6000 RCF in a microcentrifuge (Mini Spin, Eppendorf, Hamburg, Germany) and resuspended in the treatment medium. Test tubes were then inoculated at 10^7^ CFU/mL, thus initiating the lethal carvacrol treatment. The total treatment time was set to 30 min, during which aliquots were obtained every 5 min. These samples were diluted in PBS and subsequently spread on TSAYE plates. After plate incubation (48 h/37 °C), the count of survival cells was carried out in an automatic plate counter by image analysis (Analytical Measuring Systems, Protos, Cambridge, United Kingdom). Once survival curves of LmWT and evolved strains were obtained, inactivation kinetics were compared in order to evaluate the increase in resistance of LmSCar and LmLCar against carvacrol.

Following the same protocol, the heat resistance of evolved strains was assessed and compared to that of LmWT. Test tubes containing McIlvaine buffer (without carvacrol added) were incubated at 54 °C for treatment at pH 4.0; and at 58 °C for treatment at pH 7.0. Once the appropriate temperature was reached, the test tubes were inoculated with bacteria to initiate a 30 min treatment, during which aliquots were obtained every 5 min. Then, heat inactivation kinetics were analyzed to assess whether RVs to carvacrol could also exhibit cross-resistance against food processing technologies.

### 2.5. Antibiotic Susceptibility Test

An agar disk diffusion assay was conducted to test antimicrobial susceptibility according to CLSI [32,33]. Following the suggestions for fastidious bacteria [34], bacterial cultures were grown in cation-adjusted MHB supplemented with 2.5% lysed horse blood (Sigma-Aldrich). Bacterial suspensions were then spread on MHA plates supplemented with 2.5% lysed horse blood and, after 5 min at room temperature, blank disks (Ø: 6.0 mm; Thermo Scientific™ Oxoid™ Anti-microbial Susceptibility Disk Dispenser, ST6090, Waltham, MA, USA) were placed on the surface of plates and individually impregnated with the following antibiotics: 30 µg of kanamycin sulphate, 30 µg of tetracycline, 30 µg of chloramphenicol, 400 µg of nalidixic acid sodium, 5 µg of rifampicin, 30 µg of norfloxacin, 150 µg of novobiocin sodium, 10 µg of trimethoprim, 10 µg of ampicillin, and 150 µg of cephalexin (Sigma-Aldrich). These plates were incubated at 37 °C for 24 h, after which the diameters of the resulting inhibition zones were measured (paper disks included).

These antibiotics were selected in order to evaluate different cell targets and to identify structures or pathways involved in the resistance of the evolved strains that could be related to the mechanism of action of carvacrol. Limited information is provided in CLSI documents [33,34] for testing *Listeria* spp. strains. Consequently, antibiotics concentrations were chosen and adjusted according to Yehia et al. [35] and previous experiments to achieve inhibition halos higher than 20.0 mm of LmWT and thus to enhance sensitivity to detect increased resistance to antibiotics in the evolved strains.

### 2.6. Statistical Analysis

Results from phenotypic characterization were obtained from at least 3 independent experiments carried out on different working days with different bacterial cultures. MIC and MBC data correspond to the results obtained from 5 different assays. Lethal treatment curves and antibiotic susceptibility tests are displayed as the mean ± standard deviation, using Prism 4.03 software (GraphPad Software, San Diego, CA, USA). Data were compared using analysis of variance (ANOVA) and paired *t*-tests followed by post hoc Tukey with Prism 4.03 software, and differences were considered significant if *p* ≤ 0.05.

### 2.7. Whole Genome Sequencing (WGS)

Illumina technology was used to carry out whole genome sequencing (WGS) of LmWT, LmSCar, and LmLCar, on NextSeq equipment at mid-output flow, with a total of 2 × 150 cycles (Illumina; Fasteris, SA, Geneva, Switzerland). Quality control and the genetic study were carried out as described by Berdejo, Merino, Pagán, García-Gonzalo, and Pagán [17]. The quality-control-filtered paired-end reads were mapped on the reference genome sequence of *Listeria monocytogenes* EGD-e (National Center for Biotechnology Information; NCBI accession: NC_003210.1). A total of 3.66, 4.17, and 4.37 million 150 bp-reads, with an average Phred quality score of 33.07, 33.26, and 32.82, were mapped for LmWT, LmSCar, and LmLCar, respectively. The reference genome was sufficiently covered (≥100-fold coverage depth) to allow the detection of genetic changes in the strains studied. The presence of single nucleotide variants (SNVs), short insertion (Ins), deletions (Del), and structural variations (SVs) was analyzed in the evolved strains in comparison to LmWT. The resulting genome sequences were deposited in the sequence read archive (SRA) of NCBI (BioProject ID: PRJNA669703). The accession numbers of the sample data are SAMN16457448 (LmWT), SAMN30451154 (LmSCar), and SAMN30451155 (LmLCar). Finally, specific primers (Table S1) were designed to carry out PCR amplification, and Sanger sequencing was used to verify the mutations detected in the WGS analysis.

## 3. Results

### 3.1. Isolation of RVs of L. monocytogenes against Carvacrol

Once the carvacrol evolution assays with *L. monocytogenes* were performed, LmSCar (exposed to prolonged sublethal doses of carvacrol) and LmLCar (cyclically exposed to lethal treatments of carvacrol) were selected and stored in cryovials with glycerol at −80 °C for subsequent phenotypic and genotypic characterization. The evolved strains were kept and re-cultured in the absence of carvacrol to avoid phenomena of phenotypic adaption and thus to confirm the involvement of genetic modifications on their resistance changes.

Then, MIC and MBC against carvacrol for the evolved strains were determined and compared to those obtained for LmWT, with the purpose of assessing the emergence of RVs to carvacrol after both evolution protocols (Table 1).

The MIC and MBC data reveal an increase in resistance against carvacrol in both isolated strains in comparison to LmWT, although to different extents. LmSCar showed an increase in MIC from 150 µL/L (LmWT) to 175 µL/L, i.e., an increase in resistance of more than 15%, similar to that observed for MBC values (from 250 µL/L to 300 µL/L). Regarding LmLCar, the increase in resistance was even higher, from 150 µL/L to 200 µL/L for MIC and from 250 µL/L to 350 µL/L for MBC, which corresponded to a 40% increase in resistance to carvacrol.

These results demonstrated the emergence of RVs to carvacrol in *L. monocytogenes* by both prolonged exposure to sublethal doses and reiterative exposure to short lethal treatments.

### 3.2. Decreased Lethal Efficacy of Carvacrol against RVs

**Figure 1:**
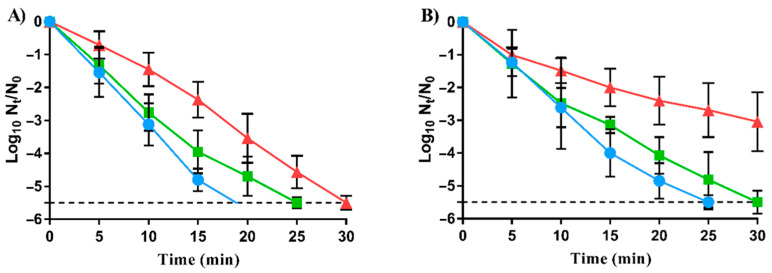
Survival curves of *Listeria monocytogenes* EGD-e (●; LmWT) and its evolved strains, LmSCar (■; exposed to prolonged sublethal doses of carvacrol) and LmLCar (▲; cyclically exposed to short lethal treatments of carvacrol), to 200-µL/L carvacrol treatment at pH 4.0 (**A**) and 300-µL/L carvacrol treatment at pH 7.0 (**B**). Data are means ± standard deviations (error bars) obtained from at least three independent experiments. The dashed line represents the detection limit (−5.5 log_10_ N_t_/N_0_).

In order to further characterize RVs, lethal carvacrol treatments were carried out under acid and neutral pH conditions. Figure 1 shows the survival curves of LmWT and its evolved strains, LmSCar and LmLCar, to lethal treatments for 30 min with 200 µL/L carvacrol at pH 4.0 (Figure 1A) and with 300 µL/L carvacrol at pH 7.0 (Figure 1B).

As shown in Figure 1A, both evolved strains exhibited a higher survival to carvacrol than LmWT under acidic conditions (*p* < 0.05). While LmWT showed inactivation greater than 5.5 log_10_ cycles at 20 min of treatment, the same level of inactivation was not achieved until 25 min of treatment by LmSCar and 30 min by LmLCar.

Regarding neutral pH, as can be seen in Figure 1B, no significant differences (*p* > 0.05) were observed between the inactivation of LmSCar and LmWT, except at 30 min. Nevertheless, the differences observed in survival at pH 7.0 between LmLCar and LmWT were greater than those observed in acid conditions (*p* < 0.05). LmWT population was reduced by more than 5.5 log_10_ cycles after 25 min of treatment, while LmLCar was only inactivated by less than 3.0 log_10_ cycles after 30 min treatment.

These results demonstrated that both evolutionary approaches could lead to the emergence of *L. monocytogenes* RVs with increased survival to carvacrol lethal doses. Nonetheless, inactivation kinetics showed that increased resistance was influenced by treatment conditions such as pH.

### 3.3. Slight Increased Cross-Resistance to Heat of RVs to Carvacrol

**Figure 2:**
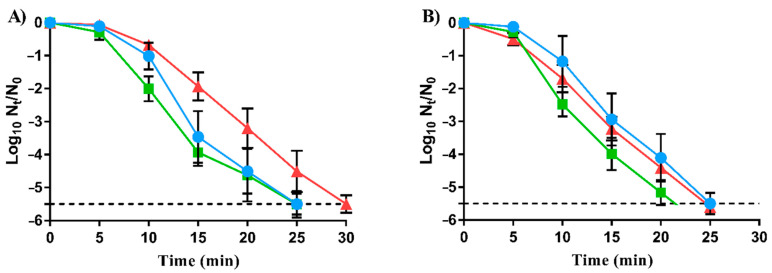
Survival curves of *Listeria monocytogenes* EGD-e (●; LmWT) and its evolved strains, LmSCar (■; exposed to prolonged sublethal doses of carvacrol) and LmLCar (▲; cyclically exposed to short lethal treatments of carvacrol), to heat treatments: 54 °C at pH 4.0 (**A**); and 58 °C at pH 7.0 (**B**). Data are means ± standard deviations (error bars) obtained from at least three independent experiments. The dashed line represents the detection limit (−5.5 log_10_ N_t_/N_0_).

The survival of carvacrol-RVs to heat treatments was characterized to assess whether evolution assays towards carvacrol resistance development can also lead to the emergence of cross-resistance to food processing methods. Figure 2 represents the survival curves of LmWT and its RVs, LmSCar and LmLCar, to heat treatments for 30 min at 54 °C/pH 4.0 (Figure 2A) and 58 °C/pH 7.0 (Figure 2B).

As illustrated in Figure 2A, no significant differences (*p* > 0.05) were observed between LmWT’s and LmSCar’s inactivation by heat at acid conditions. Only LmLCar exhibited cross-resistance against heat: LmLCar reached two log_10_ cycles of inactivation after 15 min of treatment, while LmWT and LmSCar exceeded the 3.5 log_10_ cycles (*p <* 0.05). In contrast, as shown in Figure 2B, no significant differences (*p* > 0.05) in heat survival were observed between LmWT and both carvacrol-RVs at neutral pH, except after 25 min treatment where LmSCar counts fell below the detection limit (−5.5 log_10_ cycles). It should be taken into account that different food matrices and heat treatments might lead to higher or lower differences in heat resistance between RVs and LmWT.

The slight differences in heat resistance observed (54 °C at pH 4.0 for 15 min) in LmLCar compared to LmWT might be because the mutations acquired by LmLCar during carvacrol evolution assay also provide protection against this food processing technology. These results support that carvacrol and heat may share some target structures or mechanisms of action.

### 3.4. Ampicillin Resistance Changes in RVs to Carvacrol

The occurrence of cross-resistance to other types of antimicrobials, such as antibiotics for clinical use, was also assessed in LmSCar and LmLCar. Table 2 displays the inhibition halos obtained by agar disk diffusion in LmWT, LmSCar, and LmLCar for several antibiotics: kanamycin sulfate, tetracycline, chloramphenicol, nalidixic acid, rifampicin, norfloxacin, novobiocin, trimethoprim, ampicillin, and cephalexin.

The antibiotic inhibition halos in RVs to carvacrol showed no significant differences (*p* > 0.05) with respect to LmWT for any of the antibiotics tested, except for ampicillin in both strains. However, the behavior of each carvacrol-RV against ampicillin was different (*p* < 0.05). While LmLCar showed a decrease in the ampicillin-inhibition halo size in comparison with LmWT from 20.28 mm to 18.74 mm, i.e., an increase in cross-resistance, LmSCar was more sensitive to this antibiotic showing an increase in inhibition halo of up to 23.94 mm.

### 3.5. Whole-Genome Sequencing of RVs to Carvacrol

After phenotypic characterization, the whole genomes of the evolved strains were sequenced and compared with that of LmWT to identify the mutations that could be responsible for their increased resistance against carvacrol, heat, and in the case of LmLCar, against ampicillin. We first identified several common mutations in LmWT and its RVs compared to the reference genome (NCBI accession: NC_003210.1) which were ignored (Table S2). Table 3 and Table 4 summarize the genetic modifications detected in LmSCar and LmLCar strains, respectively, compared to LmWT. Table 3 and Table 4 also show the function of the proteins encoded by the mutated genes. All these mutations were confirmed by Sanger sequencing in LmWT and RVs.

In LmSCar, two SNVs were observed compared to LmWT (Table 3). The first SNV was detected in lmo0891 (T341C), leading to the substitution of phenylalanine (Phe) by serine (Ser) at position 114. This mutation was located in the *rsbT* gene, which encodes the RsbT regulator of Sigma-B activity (σ^B^). The second SNV was identified in lmo2202 (C110A), resulting in the substitution of threonine (Thr) by asparagine (Asn) in the amino acid 37 of the 3-oxoacyl ACP synthase.

Table 4 displays the mutations detected in LmLCar compared to LmWT, two SNVs, one insertion, and one deletion; three of which resulted in protein changes:

(i) A transversion from thymine to guanine at position 287 bp (T287G) of lmo0785 led to the substitution of leucine (Leu) by arginine (Arg) at amino acid 96. The missense mutation was located in the *manR* gene, which encodes a transcriptional activator of a phosphotransferase system domain.
(ii) An insertion at position 423 bp of lmo1539 produced a reading frameshift in the transcription of a glycerol transporter.
(iii) A frameshift mutation at position 123 bp of lmo1921, which function has not been evidenced in vivo.

## 4. Discussion

EOs and ICs are known as potential food preservatives due to their strong antimicrobial and antioxidant properties, as well as their higher consumer acceptance compared to synthesized food additives [2,36]. Moreover, these natural antimicrobials are also under study as potential alternatives or enhancers to antibiotics treatment against multidrug-resistant (MDR) bacteria and the spread of AMR [12]. One of the most promising ICs as a food preservative is carvacrol due to its great antimicrobial properties against food-borne pathogens [37] and also against MDR bacteria [38]. This compound is mainly extracted from EOs of *Origanum vulgare*, *Thymus vulgaris*, and *Thymbra capitata* [39,40], and it is generally recognized as safe (GRAS) by the U.S. Food and Drug Administration [41]. However, the long-term effects of carvacrol should be studied to assess whether, as shown in antibiotics [42], RVs could also appear in food-borne pathogens such as *L. monocytogenes.*

Following evolution assays, MIC and MBC of carvacrol against LmWT, LmSCar, and LmLCar were determined (Table 1). The MIC of carvacrol obtained for LmWT in our study (150 μL/L), as well as the MBC (200 μL/L), was similar to that previously obtained by Ait-Ouazzou et al. [43] (<200 μL/L). Our result differs slightly from the MIC of 625 μg/mL obtained by Field et al. [44], probably due to differences in the methodology followed. As detailed in Table 1, the increase in resistance was higher in LmLCar, which could indicate that evolution through reiterative exposure to lethal treatments would lead to the emergence or selection of strains more resistant to carvacrol than using prolonged exposure to sublethal doses.

Although recent studies have shown that exposure to carvacrol can lead to the occurrence of these RVs in other food-borne pathogenic bacteria, to the best of our knowledge, this is the first report that shows the emergence of RVs to carvacrol in *L. monocytogenes*. Several authors have reported increased resistance to carvacrol in *E. coli* when bacteria have been exposed to prolonged sub-inhibitory concentrations of the compound, reaching up to a three-fold increase in the MIC [15,20,45,46]. This phenomenon has also been observed in *S.* Typhimurium both through exposure to sub-inhibitory concentrations and through cyclical exposure to lethal treatments. It should be noted that the evolved strain of *S.* Typhimurium by lethal treatments was more resistant than the one isolated by sublethal doses [17], as we observed in *L. monocytogenes*. In this sense, it seems that the evolution protocol using lethal treatments is more aggressive than that using sublethal doses, thus allowing the isolation of more resistant evolved strains. RVs to carvacrol have also been isolated in Gram-positive bacteria, specifically against *S. aureus* [18].

To further characterize the resistance of the evolved strains, lethal treatments with carvacrol were carried out in a citrate-phosphate buffer in acid and neutral conditions. At pH 7.0, carvacrol concentration was increased to 300 μL/L since 200 μL/L used at pH 4.0 was not enough to achieve inactivation of *L. monocytogenes* EGD-e, as was observed previously in brain–heart infusion by Field, Daly, O’Connor, Cotter, Hill, and Ross [44]. As can be observed in Figure 1, both RVs showed increased survival compared to LmWT at pH 4.0 and pH 7.0. Comparing RVs, LmSCar was more susceptible than LmLCar to lethal treatments at both pHs. Other studies have also demonstrated that strains isolated from *E. coli* [15], *S.* Typhimurium [17], and *S. aureus* [18] after evolution assays by both sublethal and lethal doses of carvacrol had increased their resistance against lethal treatments. These results support that the effectiveness of lethal treatments with carvacrol could not be enough to inactivate the target pathogen bacteria if these RVs emerge in the food chain. Furthermore, results evidenced that LmLCar likely exhibited greater survival than LmSCar because of the different evolution protocols followed in their isolation. Moreover, the inactivation kinetics in lethal treatments could explain how LmLCar could be selected after evolution assay by lethal doses of carvacrol. Once LmLCar appears by mutations, its greater survival to lethal treatments would cause its concentration in the population to be higher than that of LmWT along the cycles of the evolution assay until genetic variations of LmLCar became fixed in the bacterial population.

Heat treatments were also carried out to determine if RVs to carvacrol could pose a food safety risk by physical food preservation treatments at both acid and neutral pH. As observed in Figure 2A, only LmLCar exhibited a slight increase in heat resistance in comparison to LmWT at pH 4.0. Therefore, under these treatment conditions, LmLCar could survive lethal treatments pre-established for the reduction of LmWT. However, no significant differences were detected (*p* > 0.05) among the three strains at neutral pH (Figure 2B). According to Chueca, Berdejo, Gomes-Neto, Pagán, and García-Gonzalo [15], RVs of *E. coli* to carvacrol revealed increased heat resistance compared to the WT strain. After a 26 min treatment at 55 °C, the WT strain was inactivated by more than five log_10_ cycles, while the survival of RVs was only reduced by less than 3.5 log_10_ cycles. However, only slight differences were observed in *S. aureus* RVs to carvacrol in acid conditions [18].

Hence, it is likely that the emergence of heat cross-resistance in RVs to carvacrol depends on the bacteria under study, the evolution protocol followed, and even the conditions used in lethal treatments, such as the pH. Although laboratory conditions used to obtain the RVs of this study are different from those used in food industry settings, the occurrence of RVs in real food should be taken into account, as demonstrated by the emergence of AMR bacteria in clinical settings [8,9]. The appearance of RVs in the food chain may compromise the effectiveness of food preservation treatments. In this regard, more studies are required to determine whether the occurrence of these RVs in food matrices could pose a food safety risk by surviving food preservation treatments, such as heat, which would be *a priori* enough to inactivate them.

Antibiotic susceptibility results of the carvacrol-RVs did not reveal significant differences (*p* > 0.05) in comparison with LmWT for the majority of compounds tested. Only against ampicillin did both RVs show significant differences (*p* < 0.05) compared to LmWT. Ampicillin is a β-lactam antibiotic which acts on the binding to specific penicillin-binding proteins located inside the bacterial cell wall inhibiting the third and last stage of cell wall peptidoglycan synthesis [47]. Therefore, it is likely that the selected mutations in the carvacrol-RVs might modify the cell wall and thus lead to changes in antibiotic resistance. These results would support that the antimicrobial action of carvacrol may share cellular targets on cell envelopes with ampicillin.

Recent studies on *S.* Typhimurium RVs obtained by lethal carvacrol treatments [17] and *E. coli* RVs isolated by sublethal doses of carvacrol [20] revealed increased cross-resistance to a wide range of antibiotics, such as tetracyclines, quinolones, aminoglycosides, and beta-lactams. However, this cross-resistance to antibiotics was not observed in carvacrol-RVs of *S.* Typhimurium or *S. aureus* obtained by sublethal doses of carvacrol [16].

In this regard, it is likely that the evolution protocol and the bacterial strains may be involved in why cross-resistance to antibiotics occurs. Moreover, it is possible that carvacrol shares modes of action with more antibiotics, not just with ampicillin, depending on the bacteria under study. Nonetheless, further studies are required to find out under which conditions this cross-resistance can appear and also the relevance that increased resistance may have on clinical treatment in case these RVs infect the consumers.

Finally, WGS allowed us to detect the mutations in LmSCar (Table 3) and LmLCar (Table 4) compared to LmWT, which might be causing their increased resistance.

Two SNVs hitting different genes were detected in LmSCar. The first one was located in the *rsbT* gene, which encodes one of the regulators of sigma B (σ^B^) activity [48]. The σ^B^ controls the general stress response in Gram-positive bacteria contributing to stress tolerance by the upregulation of approximately 300 genes in the case of *L. monocytogenes*. Previous studies have demonstrated that the lack of σ^B^ resulted in decreased resistance in *L. monocytogenes* to *Origanum vulgare* and *Rosmarinus officinalis*, whose main compound is carvacrol [49], indicating the relevance of σ^B^ activity for survival against natural antimicrobials. In this regard, it can be hypothesized that the *rsbT* mutation in LmSCar could lead to an increase in σ^B^ regulon activity that provides greater protection against carvacrol.

The second SNV was detected in lmo2202, which encodes 3-oxoacyl ACP synthase. This enzyme is involved in the type II fatty acid elongation cycle, which is an essential step in the thermal regulation of fatty acid composition and, consequently, in cell wall and membrane lipid homeostasis [50]. Previous studies have pointed to cell envelopes as one of the main target structures for natural antimicrobials [51,52,53]. The mutation found in lmo2022 might alter this structure and thus provide increased carvacrol resistance to LmSCar.

Regarding LmLCar, three mutations leading to protein changes were identified in its genome. One SNV was located in the *manR* gene, encoding a positive regulator of the *manLMN* operon responsible for the transport and utilization of mannose [54]. Moreover, according to Buck et al. [55], ManR probably assists σ^54^ in melting the promoter of *mpt* during transcription initiation. Therefore, the *manR* mutation may modify the expression of its target genes, leading to changes in resistance to this antimicrobial compound. Although there is no study showing a relationship between the *manR* gene and resistance to EO or IC, several authors have linked this gene with class IIa bacteriocin resistance in *L. monocytogenes* [56,57,58].

In addition, an insertion in the *glpF1* gene of LmLCar was detected, resulting in a reading frame shift that may lead to loss-of-function of glycerol uptake facilitators. This structural component is mainly involved in extracellular glycerol diffusion across the cytoplasmic membrane via pore-type mechanisms [59]. However, there is no information on the involvement of glycerol transporters in bacterial resistance to natural antimicrobials. Since this mutation is related to the cellular metabolism of glycerol, it may be possible that this was selected during the recovery and growth steps between lethal treatments and not by the carvacrol selective pressure.

Lastly, a frameshift mutation was located in a gene (lmo1921) encoding a hypothetical protein whose function has not been evidenced in vivo. Therefore, further knowledge of the cellular function of this gene is necessary to anticipate its contribution to increased carvacrol resistance.

To sum up, WGS revealed the mutations in LmSCar and LmLCar that might be involved in their increased (cross)-resistance. Among them, it should be noted that those located in the genes encoding the transcriptional regulators RsbT (in LmSCar) and ManR (in LmLCar) are likely involved in their increased resistance to the natural antimicrobial. In LmSCar, in addition to the *rsbT* mutation, the genetic modification in lmo2202 could also contribute to its increased resistance since this gene encodes an enzyme involved in the biosynthesis of fatty acids of cell envelopes, which are one of the main targets of carvacrol. In LmLCar, in addition to the *manR* mutation, the genetic modification found in lmo1912 and *glpF1* could be involved in the development of resistance to carvacrol and also cross-resistance to heat treatments and ampicillin.

## 5. Conclusions

This study demonstrates that both the serial exposure of *L. monocytogenes* to sublethal doses and lethal doses of carvacrol could allow the emergence and isolation of genetic RVs: LmSCar and LmLCar, respectively. Both RVs exhibited an increased resistance and survival against carvacrol compared to the WT strain (LmWT), being LmLCar the most carvacrol-resistant RV. LmLCar also exhibited cross-resistance against ampicillin and heat treatments (at acid conditions), while in LmSCar, no significant increased resistance was detected, even though it was more susceptible to ampicillin than LmWT.

WGS identified two mutations in LmSCar and three mutations in LmLCar, leading to protein changes. Based on the function of the genes mutated in LmSCar, it can be hypothesized that variations in σ^B^ activity regulating the general stress response and in the ACP synthase activity involved in the cell wall synthesis might be responsible for the increased resistance to carvacrol. Regarding LmLCar, the mutation located in *manR* could be related to changes in gene expression that provide higher resistance to the natural antimicrobial. In addition, the mutation found in the lmo1921 gene of LmLCar may contribute to improving carvacrol resistance.

In brief, these results support the importance of knowing how RVs to carvacrol appear and the impact they could pose on food safety and in the clinical treatment of infections. Although the RVs of this study were obtained in laboratory growth media, the emergence of RVs in food matrices is likely to occur. As demonstrated, different genomic changes might appear in the presence of carvacrol, leading to RVs with increased resistance against food preservation treatments. Therefore, different mutations in RVs obtained in food systems would be expected with a higher or lower impact on bacterial resistance against food preservation treatments than the RVs of this study. Thus, this information will enable the design of effective preservation strategies to prevent the occurrence of RVs or to eliminate them if they do emerge in the food industry.

## Figures and Tables

**Table 1:** Minimum inhibitory concentration (MIC; µL/L) and minimum bactericidal concentration (MBC; µL/L) of carvacrol for *Listeria monocytogenes* EGD-e (LmWT) and the selected evolved strains: LmSCar (exposed to prolonged sublethal doses) and LmLCar (cyclically exposed to short lethal treatments).

Strains	MIC (µL/L) ^1^	MBC (µL/L) ^1^
LmWT	150	250
LmSCar	175	300
LmLCar	200	350

^1^ Each value represents the result of 5 different experiments carried out with different bacterial cultures of selected evolved strains on different working days.

**Table 2:** Diameters (mm) of growth inhibition halos observed in agar disk diffusion assays of *Listeria monocytogenes* EGD-e (LmWT) and its evolved strains, LmSCar (exposed to prolonged sublethal doses of carvacrol) and LmLCar (cyclically exposed to short lethal treatments of carvacrol), against antibiotics: 30 µg of kanamycin sulfate, 30 µg of tetracycline, 30 µg of chloramphenicol, 400 µg of nalidixic acid sodium, 5 µg of rifampicin, 30 µg of norfloxacin, 150 µg of novobiocin sodium, 10 µg of trimethoprim, 10 µg of ampicillin, and 150 µg of cephalexin. Each value represents the mean diameter of the inhibition halo ± standard deviation from three independent experiments.

Antibiotics	Strains		
	LmWT	LmSCar	LmLCar
Kanamycin	22.86 ± 0.99	23.20 ± 0.34	23.77 ± 1.80
Tetracycline	35.43 ± 0.76	37.99 ± 1.18	35.07 ± 0.40
Chloramphenicol	24.28 ± 0.92	25.99 ± 2.44	23.41 ± 0.97
Nalidixic acid	21.62 ± 1.22	21.21 ± 0.86	19.92 ± 1.14
Rifampicin	33.91 ± 0.97	33.71 ± 0.71	34.79 ± 1.12
Norfloxacin	22.03 ± 1.16	23.50 ± 0.43	24.52 ± 1.49
Novobiocin	31.18 ± 0.41	33.06 ± 0.51	33.38 ± 1.32
Trimethoprim	35.30 ± 1.05	34.94 ± 2.43	34.19 ± 0.44
Ampicillin	20.28 ± 0.14	23.94 ± 0.34 *	18.47 ± 0.20 *
Cephalexin	21.97 ± 1.32	25.04 ± 1.03	20.63 ± 1.61

* Significantly different (*p* < 0.05) from LmWT.

**Table 3:** Mutations of LmSCar (evolved by exposure to prolonged sublethal doses of carvacrol) in comparison with LmWT, verified by Sanger sequencing. SNV: single nucleotide variation.

Genome Position	Locus Tag	Gene	Mutation *	Change	Information
928,651	lmo0891	*rsbT*	SNV: T341C	Phe114Ser	Positive regulation of sigma-B activity
2,291,818	lmo2202		SNV: C110A	Thr37Asn	3-oxoacyl ACP synthase

* Position with respect to the start of the coding region.

**Table 4:** Mutations of LmLCar (evolved by cyclic exposure to short lethal treatments of carvacrol) in comparison with LmWT, verified by Sanger sequencing. SNV: Single nucleotide variation, Ins: insertion, and Del: deletion.

Genome Position	Locus Tag	Gene	Mutation *	Change	Information
810,506	lmo0785	*manR*	SNV: T287G	Leu96Arg	Transcriptional regulator
1,575,317	lmo1539	*glpF* *1*	Ins: +T 423	Gly142 Frameshift	Glycerol transporter
1,870,663	lmo1799		SNV: G1581T	Silent mutation (Ala527)	Peptidoglycan binding protein
1,996,626	lmo1921		Del: -T 123	Ile41 Frameshift	Hypothetical protein

* Position with respect to the start of the coding region.

## Data Availability

The original contributions presented in the study are included in the article/Supplementary Material, and further inquiries can be directed to the corresponding authors. The resulting genome sequences were deposited in the sequence read archive (SRA) of NCBI (Bio Project ID: PRJNA669703).

## Supplementary Materials

The following supporting information can be downloaded at: https://www.mdpi.com/article/10.3390/foods11203282/s1, Table S1: Primers used for PCR amplification and Sanger sequencing to verify the mutations of evolved strains: LmSCar (by cyclic exposure to prolonged sublethal doses of carvacrol) and LmLCar (by cyclic exposure to short lethal treatments of carvacrol); Table S2: Genetic variations detected by whole genome sequencing (WGS) between LmWT and the reference genome of *Listeria monocytogenes* EGD-e (NCBI accession: NC_003210.1).
