# Supplementary material for: Carvacrol Selective Pressure Allows the Occurrence of Genetic Resistant Variants of Listeria monocytogenes EGD-e
Source: Foods. 2022 Oct 20;11(20):3282. doi: 10.3390/foods11203282 (PMC9602272; doi:10.3390/foods11203282)
Supplement: Supplementary file 1 [file foods-11-03282-s001.zip › foods-1919535-supplementary.pdf]

Table S1. Primers used for PCR amplification and Sanger sequencing to verify the mutations of evolved strains, LmSCar (exposed to prolonged sublethal doses of carvacrol) and LmLCar (cyclically exposed to short lethal treatments of carvacrol).

LmSCar Mutations	Forward Primer (5′ → 3′)	Reverse Primer (5′ → 3′)
lmo0891	TTCGGCGATGGACCTTGAAA	ATCCGGCAACTCTTTTCCGT
lmo2202	CCGCACCGACAACTACGATA	ATTGACCGTCGTGCAGATGT
lmo0785	CCCCCTTAACGTGTCCGAAA	TCGCGAGCAATTAGAGCAGA
lmo1539	GAACCCATGAGTAGCCCCAA	TCCAAAGCAGAGAATGGCGG
lmo1799	CAGTCAATGTCTACATCGGCATC	CGTGGGTTTATTACTACGGATGT
lmo1921	ATCCAGCGCCTCATCGATTT	GGAAGAAGGAGAACACGCGA

Table S2. Genetic variations detected by whole genome sequencing (WGS) between LmWT and the reference genome of *Listeria monocytogenes* EGD-e (NCBI accession: NC_003210.1). Single nucleotide variation (SNV).

Genome Position	Locus Tag	Mutation*	Change	Information
264,578	lmo0247	SNV: G147T	Silent mutation (Gly49)	Hypothetical protein
435,968	lmo0412-lmo0413	SNV: G by T	Non-coding region	Hypothetical protein - hypothetical protein
966,277	lmo0929	SNV: A33G	Silent mutation (Leu11)	Sortase
1,374,715	lmo1349	SNV: A597G	Silent mutation (Leu199)	Glycine dehydrogenase subunit 1
1,442,124	lmo1412- lmo1413	SNV: C by A	Non-coding region	Modulates DNA topology – peptidoglycan binding protein
2,229,938	lmo2144	SNV: G-11A	Regulatory region	GntR family transcriptional regulator
2,943,565	lmo2855 - lmo2856	SNV: C by A	Regulatory region	Ribonuclease P – 50S ribosomal protein L34

*Position respect to the start of the coding region.
